# Online, asynchronous training in research for residents

**DOI:** 10.1017/cts.2024.631

**Published:** 2024-10-29

**Authors:** Jason T. Blackard, Jacqueline M. Knapke, Stephanie Schuckman, Jennifer Veevers, William D. Hardie, Ruchi Yadav, Alexa Kahn, Patrick Lee, Sima Terebelo, Patrick H. Ryan

**Affiliations:** 1 Division of Digestive Diseases, Department of Internal Medicine, University of Cincinnati College of Medicine, Cincinnati, OH, USA; 2 Center for Clinical and Translational Science and Training, University of Cincinnati, OH, USA; 3 Department of Family and Community Medicine, University of Cincinnati College of Medicine, Cincinnati, OH, USA; 4 Department of Pediatrics, University of Cincinnati College of Medicine, Cincinnati, OH, USA; 5 Division of Hematology and Oncology, One Brooklyn Heath, Brooklyn, NY, USA; 6 Division of Cardiovascular Medicine, Maimonides Medical Center, Brooklyn, NY, USA; 7 Department of Medicine, SUNY Downstate Medical Center, Brooklyn, NY, USA; 8 Division of Rheumatology, One Brooklyn Health, Brooklyn, NY, USA; 9 Division of Biostatistics and Epidemiology, Cincinnati Children’s Hospital, Cincinnati, OH, USA

**Keywords:** Research training, resident, scholarly activity, programme evaluation, education

## Abstract

Scholarly activity is a key component of most residency programmes. To establish fundamental research skills and fill gaps within training curricula, we developed an online, asynchronous set of modules called *Research 101* to introduce trainees to various topics that are germane to the conduct of research and evaluated its effectiveness in resident research education. *Research 101* was utilised by residents at One Brooklyn Health in Brooklyn, NY. Resident knowledge, confidence, and satisfaction were assessed using pre- and post-module surveys with 5-point Likert scaled questions, open-ended text responses, and a multiple-choice quiz. Pre-module survey results indicated that residents were most confident with the *Aligning expectations*, *Introduction to research*, and *Study design and data analysis basics* modules and least confident with the *Submitting an Institutional Review Board protocol* and *Presenting your summer research* modules. Post-module survey responses demonstrated increased learning compared to pre-module results for all modules and learning objectives (*p* < 0.0001). “This module met my needs” was endorsed 91.4% of the time. The median score for the final quiz that consisted of 25 multiple-choice questions was 23. Thematic analysis of open-ended post-module survey responses identified multiple strengths and opportunities for improvement in course content and instructional methods. These data demonstrate that residents benefit from completion of *Research 101*, as post-module survey scores were significantly higher than pre-module survey scores for all modules and questions, final quiz scores were high, and open-ended responses highlighted opportunities for additional resident learning.

## Introduction

Scholarly activity is an important aspect of the training and development of most medical professionals and occurs during summer electives, mandatory curricular activities, extracurricular research activities, and/or longitudinal research experiences. For entering medical students, the Association of American Medical Colleges outlines research-related competencies, such as quantitative reasoning, critical thinking, and written communication [1]. A 2015 meta-analysis reported that 72% of US medical students were interested in conducting research, while 31% were interested in a career that included research [2]. Students who participated in research activities while in medical school were 3.55 times more likely to be interested in research in their future careers [2]. The 2023 Association of American Medical Colleges Medical School Graduation Questionnaire reported that 84.4% of medical school graduates participated in a research project, 63.7% submitted a paper for publication, and 48.8% planned to participate in research during their careers [3].

For residents, the Accreditation Council for Graduate Medical Education requires participation in scholarly activity before the completion of training [4]. While most residency programmes have established guidelines for scholarly activities that align with accreditation requirements, only 37% reported having an organised, comprehensive research curriculum and only 70% taught skills important to research [5]. The benefits of resident research exposure are well described in the literature and include increased lifelong learning, improved patient care, increased satisfaction with training, and higher likelihood of pursuing academic careers (reviewed in [6– 8]). Nonetheless, multiple barriers exist including a lack of mentors, lack of research infrastructure, lack of trainee interest, lack of financial support, the high demand of clinical responsibilities, and the lack of research curricula.

To establish fundamental research skills and fill gaps within training curricula, we previously piloted an online, asynchronous set of modules called *Research 101* to introduce medical students to various topics that are relevant to the conduct of research [9]. Here, we evaluated the use of *Research 101* for internal medicine residents.

## Methods

Details on the creation and pilot study of *Research 101* have been described elsewhere [9,10]. This research qualified as minimal risk to participants and was exempt from most of the requirements of the Federal Policy for the Protection of Human Subjects by the University of Cincinnati Institutional Review Board.

*Research 101* modules were offered asynchronously through the online educational platform Canvas (Salt Lake City, UT). Each module consisted of learning objectives, a pre-module survey, assignments, and a post-module survey. For each module, participants completed 1) a pre-module survey before they reviewed any assignments associated with a module, 2) all assignments in a module, and 3) a post-module survey after each module. The pre-module survey included questions based on the learning objectives for each module with responses given on a 5-point Likert scale. For example, “I am confident in my ability to identify my skills as a mentee / trainee” – which corresponds to learning objective #1 for the *Aligning Expectations* module or “I am confident in my ability to describe possible barriers to an effective mentor-mentee relationship” – which corresponds to learning objective #4 for the same module. The post-module survey also included the learning objective-based questions, as well as two additional questions with yes / no / unsure as options: 1) The content of this module met my needs? and 2) Would you recommend this module to a friend if it was not a requirement?. Open-ended text fields were also included in the post-module surveys: 1) What did you like most about this module?; 2) What did you like least about this module?; and 3) If you could change one thing about this module, what would it be?

The Research Electronic Data Capture tool hosted at the University of Cincinnati was used to collect and manage all survey data [11]. For qualitative survey responses, a thematic analysis approach was utilized where data were coded to construct thematic patterns across participant responses [11]. Changes in module Likert scale scores were assessed by subtracting pre-module from post-module Likert scores, and positive differences indicated increased confidence in knowledge regarding the module content. Statistical significance in pre-post scores was evaluated using a paired *t*-test (SAS Version 9.4). For group comparisons, pooled *p*-values were reported when the test for equality of variances (Folded F) was >0.05. When the equality of variances test was <0.05, the *p*-value from the Satterthwaite method was reported.

In February 2023, internal medicine residents (postgraduate year 2) at One Brooklyn Health, including Brookdale Hospital Medical Center and Interfaith Hospital in Brooklyn, NY were registered for *Research 101* as part of an ongoing effort to improve resident scholarly activity in an inner-city safety net hospital system. Participation was required for all 71 residents. Resident progress was encouraged by a research coordinator who sent periodic reminders to those who had not yet completed the course. Resident knowledge, confidence, and satisfaction were assessed using pre- and post-module surveys with 5-point Likert scaled questions (1 – not confident, 2 – slightly confident, 3 – moderately confident, 4 – mostly confident, and 5 – 100% confident), open-ended text responses, and a multiple-choice quiz. Survey responses, assignment responses, and quiz grades were confidential. Sixty residents responded to at least one REDCap survey, while 46 completed all surveys. Participation was required and was confirmed by completion of a final quiz consisting of 25 multiple-choice questions with one correct answer per question. Participants had access to all *Research 101* content during the final quiz, feedback on incorrect responses was provided, and there was no time limit for the completion of the quiz.

## Results

Table 1 shows pre-module and post-module survey results for each module and learning objective.

**Table 1.** Pre-module and post-survey scores for *research 101* modules and learning objectives. *N* = the number of participants responding to each post-module question

Module / Question	*N*	Pre-module mean	Post-module mean
**Introduction to research /** I am confident in my ability to . . .			
1. Define research	66	3.01	3.56*
2. Identify various types of research	65	2.96	3.57*
3. Recognize the stages of clinical / translational research	66	2.76	3.52*
4. Identify the steps of the scientific method	65	2.82	3.58*
The content of this module met my needs (yes / no / unsure)			50 / 2/ 8
Would you recommend this module to a friend if it was not a requirement? (yes / no / unsure)			42 / 4/ 11
**Aligning expectations /** I am confident in my ability to . . .			
1. Identify my skills as a mentee / trainee	59	2.90	3.69*
2. Identify possible expectations of a mentee	59	2.87	3.68*
3. Identify possible expectations of a mentor	59	2.9	3.66*
4. Describe possible barriers to an effective mentor-mentee relationship	58	2.92	3.66*
The content of this module met my needs (yes / no / unsure)			50 / 0 / 5
Would you recommend this module to a friend if it was not a requirement? (yes / no / unsure)			39 / 1/ 9
**Identifying a research mentor and a research project /** I am confident in my ability to . . .			
1. Describe the characteristics of your ideal mentor	53	2.91	3.72*
2. List resources to identify an appropriate mentor and to develop a research project	53	2.87	3.70*
3. Strategize possible solutions to common barriers to an effective mentor-mentee relationship	54	2.82	3.67*
4. Understand how to write a clear and concise research project description	55	2.78	3.62*
The content of this module met my needs (yes / no / unsure)			42 / 0 / 5
Would you recommend this module to a friend if it was not a requirement? (yes / no / unsure)			36 / 1/ 9
**Introduction to human subjects research and protections /** I am confident in my ability to . . .			
1. Understand the history of human subjects research	49	2.70	3.61*
2. Define human subjects research	50	2.78	3.62*
3. List the requirements for protecting human subjects in research	49	2.78	3.67*
The content of this module met my needs (yes / no / unsure)			42 / 0 / 3
Would you recommend this module to a friend if it was not a requirement? (yes / no / unsure)			37 / 3 / 3
**Submitting an Institutional Review Board (IRB) protocol at UC /** I am confident in my ability to . . .			
1. List resources for submitting an IRB protocol for review	48	2.71	3.58*
2. List the components of an IRB submission	48	2.71	3.56*
3. Understand common issues with IRB submissions and strategize possible solutions	48	2.61	3.65*
4. List resources for the protection of human subjects, biosafety, and animal care and training on these topics	48	2.57	3.56*
The content of this module met my needs (yes / no / unsure)			38 / 1/ 3
Would you recommend this module to a friend if it was not a requirement? (yes / no / unsure)			32/ 1/ 8
**Conducting a literature search /** I am confident in my ability to . . .			
1. Describe the use of PubMed for literature searches	48	2.94	3.79*
2. Describe the use of Google Scholar for literature searches	48	2.79	3.79*
3. List resources for conducting an effective literature search	48	2.75	3.75*
The content of this module met my needs (yes / no / unsure)			41 / 0 / 1
Would you recommend this module to a friend if it was not a requirement? (yes / no / unsure)			37 / 1/ 4
**Effective writing for publication /** I am confident in my ability to . . .			
1. List the various types of publications	48	2.85	3.75*
2. Describe the elements of a manuscript	48	2.79	3.69*
3. Describe an effective abstract	48	2.85	3.71*
4. List resources for improving ones writing skills	48	2.75	3.67*
The content of this module met my needs (yes / no / unsure)			38 / 0 / 3
Would you recommend this module to a friend if it was not a requirement? (yes / no / unsure)			35 / 1 / 6
**Transparency, rigour, and reproducibility in research /** I am confident in my ability to . . .			
1. Describe the need for transparency and its impact on the research process	48	2.81	3.79*
2. Describe the need for rigour and reproducibility and their impact on the research process	47	2.72	3.74*
3. Identify approaches to enhance the transparency, rigour, and reproducibility of your research project	48	2.68	3.77*
The content of this module met my needs (yes / no / unsure)			40 / 0 / 1
Would you recommend this module to a friend if it was not a requirement? (yes / no / unsure)			35 / 1 / 6
**Study design and data analysis basics /** I am confident in my ability to . . .			
1. List different types of epidemiologic studies	46	2.92	3.70*
2. Define key characteristics and limitations of cross-sectional studies	47	2.88	3.68*
3. Define key characteristics and limitations of cohort studies	47	2.96	3.72*
4. List resources for study design and data analysis	47	2.79	3.66*
The content of this module met my needs (yes / no / unsure)			36 / 1 / 4
Would you recommend this module to a friend if it was not a requirement? (yes / no / unsure)			35 / 1 / 5
**Presenting your summer research /** I am confident in my ability to . . .			
1. Describe elements of an effective poster	47	2.70	3.57*
2. List venues for presenting research projects	46	2.62	3.57*
3. Describe elements of an effective lab meeting presentation	47	2.61	3.64*
The content of this module met my needs (yes / no / unsure)			39 / 0 / 2
Would you recommend this module to a friend if it was not a requirement? (yes / no / unsure)			34 / 1 / 6
**Evaluating the literature and presenting a journal club /** I am confident in my ability to . . .			
1. Describe strategies for reading and critiquing a scientific article	47	2.72	3.57*
2. Describe elements of an effective journal club	47	2.70	3.66*
The content of this module met my needs (yes / no / unsure)			39 / 1 / 4
Would you recommend this module to a friend if it was not a requirement? (yes / no / unsure)			34 / 1 / 5

*p < 0.0001.

A small number of participants did not complete all survey questions; thus, the number of responses is not identical across all modules. Prior to starting, residents were most confident with the

*Introduction to research* (range 2.76 – 2.96), *aligning expectations* (range 2.87 – 2.90), and *study design and data analysis basics* (range 2.79 – 2.96) modules. Residents were least confident with the *Submitting an Institutional Review Board* (range 2.57 – 2.71) and *presenting your summer research* (range 2.61 – 2.70) modules. Post-module mean scores were significantly increased for all modules and all learning objectives compared to pre-module scores (*p* < 0.0001).

As shown in Table 1, “The content of this module met my needs” was endorsed highly across all modules (91.4% “yes” responses). “No” and “unsure” responses were highest for the *Introduction to research* module and lowest for the *Conducting a literature search* and *Transparency, rigor, and reproducibility* modules. Across all modules, “Would you recommend this module to a friend if it was not a requirement?” received 81.8% “yes” responses compared to 3.32% “no” and 14.9% “unsure” responses. “No” and “unsure” responses were highest for the *Introduction to research* and lowest for the *Conducting a literature search* modules. From a high score of 25, the median final quiz score was 23 (range: 13 – 25).

Thematic analysis of open-ended text responses from the post-module surveys identified several strengths of *Research 101*. Residents noted that the video assignments were high quality and concise but also provided the right level of detail for learners new to research. The examples, cases, and resources such as external websites and documents that were provided were helpful, and learners appreciated having the material organized for them in a sequential, logical manner. The qualitative data also identified several areas for improvement within *Research 101* including not liking the longer video assignments, even more examples would be useful, and the website was difficult to navigate at times and required a lot of clicking.

## Discussion

Research training is a highly desired component of medical education and professional development. It has been suggested that resident research may improve clinical care by fostering clinical evaluation skills, clinical reasoning, and lifelong learning [12]. Early exposure to research may also increase the number of physician-scientists [12–14]. Notably, several barriers to conducting research during residency exist, including the lack of mentors, training opportunities, curricular time, background instruction, financial support, and infrastructure, as well as the pressures of significant clinical responsibilities [5].

Structured research training is important for residency programmes and is included in current ACGME requirements [4]. Moreover, residency training programmes with organized programmes/curricula, including protected time for research, were associated with increased productivity [15]. However, existing curricula may not be available across different institutes or permit modification based on individual programmatic needs [5,12]. A systematic review of resident research programmes found that the most commonly taught topics in a research curriculum (when present and reported) were research methods, scientific writing, and biostatistics, as well as literature searches, Institutional Review Board structure, and research ethics [6]. Programmes that had a research director and a structured research curriculum were associated with higher productivity.

*Research 101* was developed to provide a comprehensive and structured introduction to crucial topics in research in a highly accessible and modifiable format. The online, asynchronous format facilitates a research training infrastructure that is highly flexible and enables individualized learning and/or programme-specific adjustments. Evaluating *Research 101* in residents demonstrated significant learning as post-module survey scores were significantly higher than pre-module scores for all modules and all learning objectives. Final quiz scores were also quite high on average. A systematic review of resident research curricula found that evaluation methods were often rudimentary, infrequently reported obstacles encountered or modifications made to the curriculum, and rarely reported pre–post-intervention testing of learners’ knowledge [12]. Thus, we consider the evaluative component of *Research 101* to be a major strength.

This study has several limitations of note. First, *Research 101* was piloted initially with a small number of medical students and then expanded later to include additional learner types such as residents. We recognize that distinct research educational needs are likely based on learner type and that the modules within *Research 101* may require adaptation to these specific needs in subsequent iterations. Second, individuals who participated may have research interests or had prior research experiences that may contribute to selection bias amongst those completing *Research 101*. While not available for this iteration, the subsequent version of *Research 101* now includes demographic data, as well as responses to the questions 1) have you ever published a peer-reviewed manuscript and 2) are you currently involved in the conduct of research? Third, self-efficacy/knowledge was not assessed using a standardized instrument and instead utilized a non-validated scale. Fourth, the fact that completion of *Research 101* was required by the residency programme directors may have unduly influenced the findings reported here. Fifth, although *Research 101* is offered asynchronously, several topics included would benefit from more direct interactions with learners. Thus, we must emphasize that *Research 101* does not replace in-person interactions; rather, it provides an additional option for research education given the limited space for new content within existing resident curricula while also acknowledging the varying learning systems of learners.

*Research 101* provides a comprehensive overview of research-relevant topics that complement the learning environment for residents who are preparing for or already conducting research projects. It can serve as an important addition to the research training toolkit with clear evidence of enhanced learning and confidence in research-related understanding.
